# 18-Fluorodeoxyglucose Uptake by Positron Emission Tomography in Extraocular Muscles of Patients with and without Graves' Ophthalmology

**DOI:** 10.1155/2013/529187

**Published:** 2013-02-14

**Authors:** Leonardo García-Rojas, Gloria Adame-Ocampo, Erick Alexánderson, José Luis Tovilla-Canales

**Affiliations:** ^1^Instituto de Oftalmología “Fundación de Asistencia Privada Conde de Valenciana I.A.P.”, 06800 Mexico City, DF, Mexico; ^2^Unidad PET/CT Ciclotrón, Facultad de Medicina, Universidad Nacional Autónoma de México, 04510 Mexico City, DF, Mexico

## Abstract

*Objective*. To compare 18-fluorodeoxyglucose (FDG) uptake by positron emission tomography (PET) in extraocular muscles (EOMs) of patients with Graves' ophthalmopathy (GO) versus patients without GO. *Design*. Prospective, observational, comparative, and cross-sectional study. *Participants*. Thirty-two eyes of patients with GO and seventy eyes of patients without GO. *Methods*. We prospectively included patients older than 18 years of age with and without GO. FDG-PET imaging study was performed; standardized unit value (SUV_max_) was quantified in EOMs. Standard deviation and significant statistical difference (*P* < 0.05) were calculated. *Results*. Thirty-two eyes of sixteen patients of the GO group were included, with a mean age of 44.31 (20–71) years. Seventy eyes of thirty-five patients of the group without GO were included, with a mean age of 49.20 (24–77) years. EOMs average uptake of the groups with and without GO were 3.38 ± 1.31 and 1.89 ± 0.51 SUV_max_ (*P* < 0.05), respectively. *Conclusion*. FDG uptake was significantly increased in EOMs of patients with GO. PET gives valuable information and may be a helpful tool in detecting, localizing, and quantifying GO inflammation. Further research is needed to define the role of PET in detecting, grading, and following up GO in order to optimize treatment in the inflammatory stage.

## 1. Introduction

Graves' disease is one of the most common autoimmune illnesses with an annual incidence in women of 1 in 1000 in the general population [1]. In addition to thyroid involvement, Graves' disease develops thyroid-associated ophthalmopathy in 25% to 50% of patients [2]. The annual incidence of Graves' ophthalmopathy (GO) is 16 in every 100,000 women and 3 in every 100,000 men [3]. Despite the fact that the majority of cases of GO are mild, approximately 5% represent severe cases with chemosis, ptosis, and visual disturbances, among others [1].

Physiopathogenic theories of GO have pointed to the inflammation of the orbital tissue generated by the immunoreactivity of the thyroid-stimulating hormone receptors (TSHR) [1, 4]. Inflammation and lymphocytic infiltration of fibroblasts are generated by the presence of anti-TSHR antibodies and anti-insulin growth factor receptor 1 (IGF-1R) [1, 5].

The expansion of the orbital tissues shifts the eyeball forward; this prevents the orbital venous outflow. These changes, combined with local production of cytokines and other inflammation mediators, tend to generate pain, proptosis, periorbital edema, conjunctival injection, and chemosis [1]. However, the anatomical variability of the orbits and the complex interaction of pathogenic mechanisms pose a real challenge to make a reproducible, clinically useful, and precise classification so as to make proper diagnostic and therapeutic decisions [6]. For this reason, diagnosis is enriched and sometimes clarified when it is supported by imaging studies. Diagnostic imaging methods such as CT show an increased volume in extraocular muscles (EOMs) and in orbital fat in a simultaneous or isolated manner, although it has been found that, in its early stages, EOMs do not show significant changes [1]. Structural changes in GO are the result of inflammatory changes. It is a dynamic process that requires a functional and structural study capable of assessing with greater certainty the type and degree of orbital condition of this disease.

Positron emission tomography (PET) is a noninvasive diagnostic method that has been proposed as a mean for detecting inflammatory and/or neoplastic processes. PET offers the ability to perform functional and metabolic assessment in cases of the presence or absence of any structural alteration [7]. One of its main advantages over other methods is its ability to detect early inflammatory stages prior to structural changes in the tissue [8]. 18F-fluorodeoxyglucose (FDG) is a radionuclide molecular analogue of glucose that is being used as a metabolic marker. The difference in their concentrations is the basis for diagnosis with PET and is given by a greater cellular uptake of glucose [9]. This method has the advantage of being “semiquantitative” according to the concentration of the radionuclide by the means of the standardized units of value (SUV) per gram of the tissue being studied, in relation to patient's weight and amount of the radionuclide being administered. This tool facilitates the determination of normal and pathologic uptake of the radionuclide in the tissues under study without subjective interpretation [10].

Lymphocytes had shown high affinity to FDG [11–13], a very sensitive fact associated with inflammation. Inflammatory processes and malignant diseases are the most significant cause of FDG uptake. Glycolytic metabolism in inflammatory processes is elevated due to leukocyte infiltration and metabolism.

The general objective of this study is to compare FDG uptake by PET in extraocular muscles of patients with Graves' ophthalmopathy (GO) versus patients without GO.

## 2. Materials and Methods

This prospective, observational, and cross-sectional study was carried out in the Thyroid Clinic of the “Conde de Valenciana” Ophthalmology Institute and the PET/CT Unit from the Medicine School of the “Universidad Nacional Autónoma de México” (National Autonomous University of Mexico). The study was approved by the Institutional Review Board and Ethics Committees and conducted according to the tenets of the Declaration of Helsinki. We included patients of 18 years and older. GO group included patients who suffered from GO with some degree of clinical inflammatory activity, whether mild, moderate, or severe without anti-inflammatory treatment or at least six months of having discontinued it. Patients without GO included patients who underwent an FDG-PET study due to another indication different from orbital, ocular, and thyroid pathology and without the usage of anti-inflammatory medication. All of them underwent an FDG-PET of orbital structures with a protocol of minimal cerebral, visual, and ocular motility stimulation. 18-FDG uptake of PET images was analyzed in terms of maximum standardized unit values (SUV_max⁡_). The first group included all eyes from patients with GO, while the second group included all eyes without GO. These groups were compared according to the mean value of FDG uptake in SUV_max⁡_ and analyzed in order to determine whether the difference in the value of FDG uptake was significant or not. Statistical analysis was performed with Student's *t*-test and SPSS (v. 17; SPSS Inc., Chicago, IL, USA).

## 3. Results

We included thirty-two eyes of sixteen patients, 10 women and 6 men, with a mean age of 44.31 ± 13 years (range 20–71) from the GO group. From the group without GO, seventy eyes of thirty-five patients, 18 women and 17 men, with a mean age of 49.20 ± 14.36 years (range 24–77) were included.

**Figure 1:**
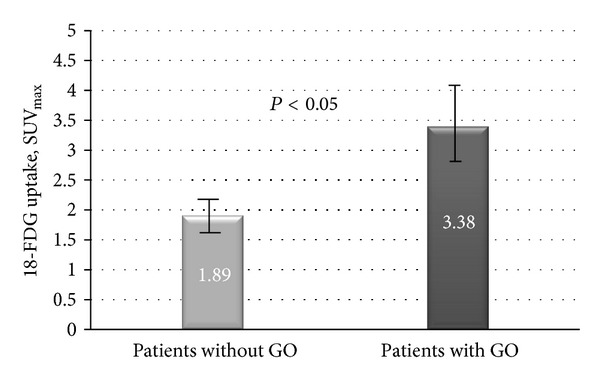
Mean and standard error extraocular muscle FDG uptake in patients without and with GO (1.89 ± 0.51 and 3.38 ± 1.31  SUV_max⁡_, resp.).

Mean values of muscle FDG uptake were compared as a whole (superior, inferior, medial, and lateral recti and superior oblique) and separately between both groups. Mean extraocular muscle uptake of the GO group was 3.38 ± 1.31 SUV_max⁡_, while the mean value of the group without GO was 1.89 ± 0.51 SUV_max⁡_ (*P* < 0.05) (Figure 1).

**Figure 2:**
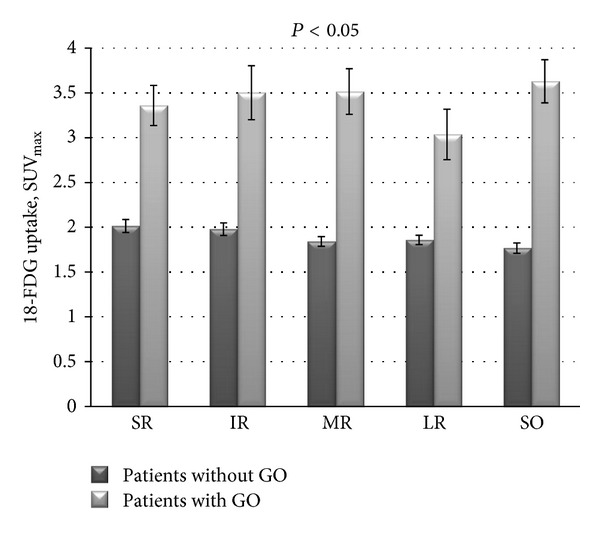
Mean and standard error extraocular muscle FDG uptake of patients without and with GO. SR: superior rectus, IR: inferior rectus, MR: medial rectus, LR: lateral rectus, and SO: superior oblique.

Values of mean uptake of each extraocular muscle of both groups were as follows: superior rectus (SR) 3.36 ± 1.23 versus 2.01 ± 0.61  SUV_max⁡_ (*P* < 0.05), inferior rectus (IR) 3.50 ± 1.71 versus 1.98 ± 0.58  SUV_max⁡_ (*P* < 0.05), medial rectus (MR) 3.52 ± 1.44  versus 1.84 ± 0.45  SUV_max⁡_ (*P* < 0.05), lateral rectus (LR) 3.04 ± 1.55 versus 1.86 ± 0.44  SUV_max⁡_ (*P* < 0.05), and superior oblique (SO) 3.63 ± 1.23  versus 1.77 ± 0.48  SUV_max⁡_ (*P* < 0.05), of patients with and without GO, respectively. Inferior oblique values were impossible to determine due to its path and localization (Figure 2).

The muscles with the lowest and highest FDG uptake from the GO group were the lateral and inferior recti with 0.9 and 8.2 SUV_max⁡_, respectively. The lowest and highest values for the group without GO were the superior oblique and lateral rectus with 0.8 SUV_max⁡_ and the superior and inferior rectus with 2.9 SUV_max⁡_.

Physiological FDG accumulation in extraocular muscles—except for inferior oblique—of patients without GO is described in Table 1.

**Figure 3:**
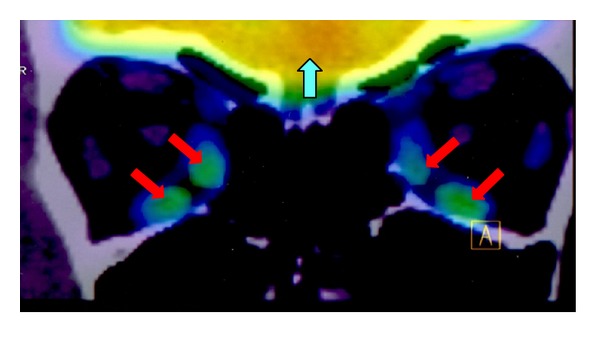
PET/CT coronal view of extraocular muscles. Warm to cold color scale (red, orange, yellow, green, blue, and purple) shows a high to low FDG uptake (accumulative radioactivity). The image shows high FDG uptake from brain (blue arrow) and an increased FDG uptake in inferior and medial recti muscles (red arrows).

As part of the GO group, PET/CT showed an increased FDG uptake in the MR and IR (3.52 and 3.50 SUV_max⁡_, resp.) (Figure 3), which are muscles that are usually thicker in patients with GO. An interesting finding in the same group is that the SO muscle showed a greater uptake than the rest of the muscles, with a value of 3.63 SUV_max⁡_, which does not correlate with previous reported increased muscle thickness in the CT evaluation.

## 4. Discussion

In 2006, Kuo et al. [14] were the first to describe a PET/CT study in a patient with GO with the same biomarker as this study. In this case, the detection of inflammation by FDG uptake was sensitive and objectively demonstrable by this semiquantitative imaging method. In this study, we screened thirty-two eyes from sixteen patients with a GO and seventy eyes from thirty-five patients without GO to compare FDG uptake results. Mean extraocular muscle uptake of the GO group was 3.38 ± 1.31SUV_max⁡_, while mean value of the group without GO was 1.89 ± 0.51 SUV_max⁡_ (*P* < 0.05). 

Normal FDG uptake of extraocular muscles has not been described in a detailed pattern. Normal values should be established in order to make possible identification of pathological processes. Inflammation of orbital tissues may be clinically difficult to identify due to the variability of its manifestation and the presence of confounding variables that may be associated to the states of noninflammatory fibrosis in the GO.

In 2005, Burrell and van den Abbeele described that FDG uptake is frequently seen at the apex and may also be seen throughout the length of extraocular muscles [15]. Later in 2007, Zincirkeser et al. [16] mentioned from Uematsu et al. [17] that extraocular muscles, the muscles of the oral cavity and the laryngeal muscles, accumulate FDG to varying degrees. In 2009, Nakamura et al. described physiological high FDG accumulation in extraocular muscles and fixated an optimal cutoff value of 10.0 SUV_max⁡_ with a left to right ratio of less than 1.5 [18]. However, values were not individualized for each extraocular muscle.

This study suggests that the sensitivity of FDG-PET/CT in detecting inflammation in GO is possibly superior to other imaging studies such as CT alone and even magnetic resonance imaging, as previous studies have reported in other structures [19]. FDG-PET/CT may be useful in assessing inflammatory status when clinical and/or serological doubt exists. PET/CT may be useful as an accurate imaging method to characterize not only morphological but also inflammatory orbital findings in patients with GO.

A limitation of this study is the sample size of the group of patients with GO; a greater sample, similar to the group of patients without GO, would be better to compare.

As a conclusion, this is the first prospective study that describes physiological FDG extraocular muscle uptake values and compares its results with patients suffering Graves' Ophthalmopathy. FDG uptake of extraocular muscles was statistically different (*P* < 0.05) between patients with and without GO. Nowadays, there is no accurate and objective tool for its clinical evaluation and for differentiating active inflammatory states from fibrosis. The clinical and serological evaluation is sometimes insufficient to determine the presence of active inflammation. In such scenarios, more and better information is needed to establish the presence of inflammation and make the best treatment decision. PET/CT provides valuable and useful information for the diagnosis, characterization, and therapeutic decision in cases where clinical doubt exists. Further research is required to define the role of FDG-PET/CT in the detection, clinical and serological correlation, classification, and monitoring of GO.

## Figures and Tables

**Table 1:** Physiological FDG uptake of extraocular muscles.

Muscle	Average	SD	Range	
SR	2.01	0.61	1.41	2.62
IR	1.98	0.58	1.40	2.56
MR	1.84	0.45	1.39	2.29
LR	1.86	0.44	1.42	2.29
SO	1.77	0.48	1.29	2.24
Average	**1.89**	**0.52**	**1.37**	**2.41**

SR: superior rectus, IR: inferior rectus, MR: medial rectus, LR: lateral rectus, and SO: superior oblique.
